# Unilateral Pulsatile Tinnitus and Intractable Vertigo as a Manifestation of Natural Gas Toxicity

**DOI:** 10.1002/oto2.76

**Published:** 2023-09-20

**Authors:** Kyle W. Leonard, Ravi R. Shah

**Affiliations:** ^1^ Department of Otolaryngology–Head and Neck Surgery Henry Ford Health System Detroit Michigan USA; ^2^ Department of Surgery Michigan State University East Lansing Michigan USA

**Keywords:** natural gas, tinnitus, toxicity, vertigo

Natural gas toxicity is known to cause a variety of neurologic and pulmonary symptoms. To date, there are no documented cases of true vertigo caused by natural gas exposure alone. We describe the case of a 34‐year‐old who developed intractable vertigo and unilateral pulsatile tinnitus with complete resolution of symptoms after addressing a gas leak in his home. We hypothesize a mechanism for this clinical presentation as it relates to natural gas toxicity. This case report was considered not to be human subjects research by the Henry Ford Health Institutional Review Board.

## Case

A healthy 34‐year‐old male presented to the otolaryngology clinic with intractable vertigo and pulsatile tinnitus that began days after firearm training, which was the initial suspected cause. He had a long history of occupational noise exposure from firearms and used hearing protection without any previous issues. He was treated by his primary care provider with a short course of low‐dose prednisone and meclizine with initial improvement followed by symptom relapse. His vertigo symptoms were a room‐spinning sensation, and the pulsatile tinnitus was entirely right‐sided and synchronous with his heartbeat. The symptoms fluctuated in severity but never resolved. Due to symptom severity, he took leave from work and remained at home. Vital signs were within normal limits, and pulse oximetry at a visit with his primary care provider for these symptoms showed a saturation of 97%. His physical exam was normal, and audiometry showed normal hearing with type A tympanogram ruling out hearing loss as a precipitant of the tinnitus especially given his recent and chronic noise exposures. Comprehensive balance function testing was normal, ruling out vestibular organ disorders. Magnetic resonance imaging (MRI) showed mild prominence of the cerebrospinal fluid (CSF) spaces in the optic nerve sheaths bilaterally but no other abnormalities, ruling out lesions of the internal auditory canal or labyrinth. He was diagnosed with vestibular migraine, though his symptoms persisted for months and did not respond to abortive therapy.

Later in his clinical course, his partner moved into his residence and developed similar symptoms. The patient surmised an environmental cause, carefully inspected his home, and noticed the faint odor of natural gas. He notified his utility company which identified and repaired two gas leaks in his home. Days later, the patient and his partner's symptoms completely resolved.

## Discussion

Natural gas has many components, predominantly methane, and incomplete combustion can produce carbon monoxide.^1^ In excess, methane has been reported as toxic by means of pneumonitis and asphyxiation.^2^ Carbon monoxide exposure is also known to cause many symptoms including dizziness and hearing loss.^3^ Reports have described cases of dizziness and hearing loss attributed to carbon monoxide exposure after symptoms resolved following home repairs.^4^ However, pulsatile tinnitus as a dominant symptom of carbon monoxide exposure is not well‐documented, and there are no reports of tinnitus or true vertigo associated with isolated natural gas exposure.

Though the mechanism is unclear, we hypothesize that the pathophysiology of this patient's presentation is similar to that of carbon monoxide toxicity or could be from carbon monoxide itself. Hypoxemia from asphyxiation can stimulate vasodilation and increase cerebral blood flow, causing intracranial hypertension, a presumed cause of pulsatile tinnitus.^5^ Given the MRI findings of increased optic nerve sheath CSF prominence, we believe this patient's natural gas exposure caused hypoxemia, subsequent intracranial hypertension, and resultant intractable pulsatile tinnitus and vertigo.

We acknowledge that any of the components in natural gas could have precipitated hypoxemia or other toxic effects, causing the patient's symptoms; however, carbon monoxide is the most likely culprit. Further testing such as ambulatory pulse oximetry or CO‐oximetry was not pursued after symptoms resolved, so we cannot prove the exact culprit or pathophysiology. We recognize that our proposed mechanism cannot be ethically studied in vivo; nonetheless, this case illustrates the importance of considering toxic exposure in patients with intractable vertigo.

## Conclusion

A 34‐year‐old male experienced intractable vertigo and unilateral pulsatile tinnitus associated with exposure to a gas leak. His symptoms were likely due to natural gas toxicity, although the exact pathophysiology is unclear. The mechanism may be similar to carbon monoxide poisoning, and perhaps from carbon monoxide itself. We hypothesize that hypoxemia from the natural gas exposure caused cerebral vasodilation and subsequent intracranial hypertension, precipitating pulsatile tinnitus and vertigo.

## Authors Contributions

**Kyle W. Leonard**, chart review for data acquisition, analysis, and manuscript planning, drafting and revising the manuscript, final review and approval of the version to be published, agreement to be held accountable for the content of this manuscript; **Ravi R. Shah**, contributions: chart review for data acquisition, analysis, and manuscript planning, revising the manuscript, final review and approval of the version to be published, agreement to be held accountable for the content of this manuscript.

## Disclosures

### Competing interests

None.

### Funding source

None.
